# Renal Ischemia Induces Epigenetic Changes in Apoptotic, Proteolytic, and Mitochondrial Genes in Swine Scattered Tubular-like Cells

**DOI:** 10.3390/cells11111803

**Published:** 2022-05-31

**Authors:** Kamalnath S. Rajagopalan, Logan M. Glasstetter, Xiang-Yang Zhu, Roman Thaler, Hui Tang, Kyra L. Jordan, Ishran M. Saadiq, Sandra M. Herrmann, Alejandro R. Chade, Maria V. Irazabal, Lilach O. Lerman, Alfonso Eirin

**Affiliations:** 1 Division of Nephrology and Hypertension, Mayo Clinic, Rochester, MN 55901, USA; sankaranrajagopalan.kamalnath@mayo.edu (K.S.R.); logan.glasstetter@duke.edu (L.M.G.); zhu.xiangyang@mayo.edu (X.-Y.Z.); tang.hui@mayo.edu (H.T.); jordan.kyra@mayo.edu (K.L.J.); saadiq.ishran@mayo.edu (I.M.S.); herrmann.sandra@mayo.edu (S.M.H.); irazabalmira.maria@mayo.edu (M.V.I.); lerman.lilach@mayo.edu (L.O.L.); 2 Department of Orthopedic Surgery, Mayo Clinic, Rochester, MN 55901, USA; thaler.roman@mayo.edu; 3 Department of Physiology and Biophysics, Medicine and Radiology, University of Mississippi Medical Center, Jackson, MS 55901, USA; achade@umc.edu

**Keywords:** renal ischemia, renal artery stenosis, mitochondria, scattered tubular-like cells, epigenetics

## Abstract

Background: Scattered tubular-like cells (STCs) are dedifferentiated renal tubular cells endowed with progenitor-like characteristics to repair injured parenchymal cells. STCs may be damaged and rendered ineffective by renal artery stenosis (RAS), but the underlying processes remain unclear. We hypothesized that RAS alters the epigenetic landscape on DNA and the ensuing gene transcriptional profile of swine STCs. Methods: CD24+/CD133+ STCs were isolated from pig kidneys after 10 weeks of RAS or sham (*n* = 3 each) and their whole 5-methylcytosine (5mC) and 5-hydroxymethylcytosine (5hmC) profiles were examined by 5mC and 5hmC immunoprecipitation sequencing (MeDIP-/hMeDIP-seq, respectively). A subsequent integrated (MeDIP/hMeDIP-seq/mRNA-seq) analysis was performed by comparing all online available gene sets using Gene Set Enrichment Analysis. Apoptosis, proteolysis, and mitochondrial structure and function were subsequently evaluated in vitro. Results: Differential expression (DE) analysis revealed 239 genes with higher and 236 with lower 5mC levels and 275 genes with higher and 315 with lower 5hmC levels in RAS-STCs compared to Normal-STCs (fold change ≥1.4 or ≤0.7, *p* ≤ 0.05). Integrated MeDIP-/hMeDIP-seq/mRNA-seq analysis identified several overlapping (DE-5mC/mRNA and DE-5hmC/mRNA levels) genes primarily implicated in apoptosis, proteolysis, and mitochondrial functions. Furthermore, RAS-STCs exhibited decreased apoptosis, mitochondrial matrix density, and ATP production, and increased intracellular amino acid concentration and ubiquitin expression. Conclusions: Renal ischemia induces epigenetic changes in apoptosis-, proteolysis-, and mitochondria-related genes, which correlate with alterations in the transcriptomic profile and corresponding function of swine STCs. These observations may contribute to developing novel targeted interventions to preserve the reparative potency of STCs in renal disease.

## 1. Introduction

Renal artery stenosis (RAS), commonly due to atherosclerosis, is a common cause of renal failure in the elderly population [1] frequently detected incidentally in patients undergoing coronary angiography [2]. Patients with RAS are prone to develop renovascular hypertension and renal dysfunction, which aggravate cardiovascular disease and increase morbidity and mortality [3]. Alas, a better understanding of the mechanisms triggering renal injury in RAS may help develop novel strategies to attenuate renal functional deterioration.

Several injurious pathways are activated in the stenotic kidney, including the renin-angiotensin-aldosterone system, renal inflammation, and microvascular remodeling [4,5]. Renal ischemia in RAS may also impair endogenous repair in the stenotic kidney [6], which partly relies on the recruitment of scattered tubular-like cells (STCs), a dedifferentiated phenotype that can be adopted by surviving tubular epithelial cells to reconstitute the tubular epithelium [7]. These cells co-express the cell surface markers CD133 and CD24 [8,9], as well as markers of proximal tubule dedifferentiation, such as vimentin [10] and kidney injury molecule (KIM)-1 [9]. We have previously shown that experimental RAS impairs the reparative potency [6,11] and the ability of STCs to preserve the structure and function of ischemic mouse kidneys [12]. However, the exact mechanisms of RAS-induced STC dysfunction remain unknown.

Epigenetic changes, defined as alterations in gene expression that do not change the DNA sequence, have been implicated in the pathogenesis of renal ischemia and play a key role in governing the phenotype of renal tubular cells [13,14,15]. Regulatory element activity, such as promoter and enhancer activity, can be influenced by epigenetic changes, such as methylation and hydroxymethylation of the carbon-5 of cytosine (5mC and 5hmC, respectively) [16,17] on DNA. 5mC is broadly accepted as a repressive epigenetic mark that hinders the binding of transcription factors, favoring the recruitment of co-repressor complexes to methylated target promoters [18]. Contrarily, 5hmC is commonly associated with transcriptional activation, which is partly achieved by modulating chromatin accessibility of the transcriptional machinery or by inhibiting repressor binding [19]. Therefore, 5mC and 5hmC may exhibit reciprocal changes in response to an altered milieu. However, whether RAS alters the 5mC and 5hmC profiles of STCs remains to be elucidated.

In this study, we compared the genomic-wide mapping of 5mC and 5hmC patterns between normal- and RAS- swine STCs using methylated and hydroxymethylated DNA immunoprecipitation combined with deep sequencing (MeDIP- and hMeDIP-seq, respectively). We hypothesize that RAS alters the epigenetic landscape and the ensuing gene transcriptional status and function of swine STCs.

## 2. Materials and Methods

### 2.1. Experimental Design

We studied 6 female domestic pigs (Manthei Hog Farm, Elk River, MN, USA) over 10-weeks. At baseline, animals were anesthetized with intramuscular tiletamine hydrochloride/zolazepam hydrochloride (5 mg/kg, Telazol^®^, Fort Dodge Animal Health, New York, NY, USA) and xylazine (2 mg/kg), and anesthesia was maintained with ketamine (0.2 mg/kg/min) and xylazine (0.03 mg/kg/min) to induce unilateral RAS in 3 pigs by placing a local irritant coil in one of the main renal arteries under fluoroscopy (RAS group). This intervention gradually develops hemodynamically significant RAS in 7–10 days [20,21,22]. The other 3 pigs underwent a sham procedure under fluoroscopy without placement of an irritant coil (Normal group).

Ten weeks later, all animals were similarly anesthetized, and renal angiography with contrast was performed to assess the degree of stenosis. Multi-detector computed tomography (MDCT) was performed to determine single-kidney hemodynamics and function, including cortical and medullary volumes, perfusion, renal blood flow (RBF), and glomerular filtration rate (GFR), and data were analyzed using Analyze (Biomedical Imaging Resource, Mayo Clinic, Rochester, MN, USA) and MATLAB 7.10 (MathWorks) [23,24]. Blood pressure was measured during MDCT using an intra-arterial catheter, and systemic blood samples were collected to measure serum creatinine levels (Gamma-Coat kit; DiaSorin, Stillwater, MN, USA).

A few days after the MDCT studies, pigs were euthanized with sodium pentobarbital (100 mg/kg IV, Fatal-Plus, Vortech Pharmaceuticals, Dearborn, MI, USA), and kidneys were harvested and dissected immediately using a retroperitoneal incision. STCs were isolated, characterized, and cultured, and their DNA and mRNA were isolated for MeDIP-, hMeDIP-, and mRNA-seq studies.

### 2.2. STC Isolation and Characterization

Swine STCs were isolated as previously described [6,11,25]. In brief, cortical and medullary sections of the kidneys were washed with 5 mL of phosphate-buffered saline, diced, and digested with 2 mg/mL of collagenase for 1 h. The fibrous component of the kidney tissue was removed with a 60-mesh steel filter (250 µm), and processed samples were passed through a 100 μm cell filter [26]. STCs were then cultured in Medium 199 with 3% FBS (Gibco BRL, Waltham, MA, USA) at 37 °C with 5% CO_2_; [27]. Non-adherent cells were removed by replacing the culture medium (Medium 199 with 3% FBS) every 2 days. Two weeks later, adherent cells were treated with trypsin (TrypLE™ Express Gibco BRL, Waltham, MA, USA) and subcultured. Immunofluorescence staining and flow cytometry were performed to characterize cultured STCs by the co-expression of CD133 (Novus Biologicals, Centennial, CO, USA) and CD24 (Abcam, San Francisco, CA, USA), and expression of vimentin (Abcam) and KIM-1 (R&D Systems, Minneapolis, MN, USA) [6].

### 2.3. MeDIP- and hMeDIP-seq

MeDIP and hMeDIP-seq were performed as previously described [28]. DNA was extracted from STCs using the DNeasy Blood and Tissue kits (Qiagen, Cat. 69504) with RNase treatment following the manufacturer’s instructions. DNA was quantitated by a nano-drop instrument and diluted into a concentration of 100 ng/μL with TE buffer. The aliquot (100 μL) of diluted gDNA was sonicated using the Bioruptor^®^ Pico (Diagenode, Seraing, Belgium) for 7–10 cycles of 30 s on and 30 s off. The size of fragmented DNA was analyzed by the fragment analyzer (Advanced Analytical Technologies, Ankeny, IA, USA) using the High Sensitivity NGS Fragment Analysis Kit (Cat. DNF-486). Fragmented DNA with an average size of 200 bp was denatured at 95 °C for 10 min. Then, 2.5–5 µg of DNA in 1× DIP buffer (10 mM sodium phosphate, pH 7.0, 140 mM NaCl, 0.05% Triton X-100) was incubated with 1µg of anti-5mC antibody (Diagenode, Cat. C15200081, clone 33D3) or anti-5hmC antibody generated from the hybridoma clone EDL HMC 1A (Millipore, Cat. MABE1093) for 3 h at 4 °C on a rotator. Protein-G Dynabeads (Thermo Fisher, Cat. 10003D) were added, and the reactions were further incubated at 4 °C on a rotator overnight. Beads-antibody-DNA complexes were extensively washed by DIP buffer and TE buffer, and enriched DNA fragments eluted from the beads, purified with the ssDNA/RNA Clean and Concentrator Kit (Zymo Research, Cat. D7010), and quantified using the Qubit ssDNA High Sensitivity assay (Thermo Scientific, Cat. Q10212). Libraries were prepared (ACCEL-NGS^®^ 1S Plus DNA Library kit, Swift Bioscience, Cat. 10024) [29] following the manufacturer’s instructions and sequenced to 51 base pairs from both ends on an Illumina HiSeq4000 instrument in the Mayo Clinic Medical Genomics Facility.

Bioinformatic analysis was performed by aligning paired-end sequenced FASTQ files to the pig reference genome using bowtie2 2.3.3.1 [30]. Duplicates were removed (PICARD 1.67, MarkDuplicates) and peaks were identified using MACS2 [31]. Differential peak analysis was performed to determine sites of differential 5mC and 5hmC coverage using the DiffBind 2.14.0 application package [32] and the HOMER 4.10 [33] peak annotation tool. Subsequent 5mC and 5hmC coverage analyses applied per-base coverage of regions-of-interest (Bedtools 2.20.0, genomeCoverageBed) and sequence read values for the overall exonic 5mC and 5hmC coverage per gene were calculated (htseq-count 0.9.1) [34]. Faux-RNA counts of 5mC and 5hmC coverage were processed (edgeR 3.28.1) [35] to determine the differences in the read frequencies for genomic 5mC and 5hmC coverage at exons analogous to differential expression (DE) analysis of RNA reads. The Benjamini–Hochberg–Yekutieli procedure was used to correct *p*-values. Heat maps of genes, according to 5mC and 5hmC levels in RAS-STCs versus Normal-STCs, were generated using Morpheus (https://software.broadinstitute.org/morpheus/, accessed on 17 February 2022).

The 5mC and 5hmC profiles of candidate genes (*ODF3B*, *TP23*, *CLDN11*, and *ZAR1*) were visualized using the Integrative Genomics Viewer (IGV) [36]. Gene ontology (GO) analysis of the cellular component, molecular function, and biological process of genes with altered 5mC and 5hmC levels in RAS-STCs versus Normal-STCs was performed using Gene Set Enrichment Analysis (GSEA version 4.0.3, Broad Institute) [37] and categories ranked based on the number of genes in overlap.

### 2.4. mRNA-seq and Integrated (MeDIP- and hMeDIP-seq/mRNA-seq) Analysis

To explore whether RAS also elicited long-lasting effects on gene transcription, mRNA-seq analysis was performed in the same cultured swine STCs, followed by an integrated (MeDIP- and hMeDIP-seq/mRNA-seq) analysis.

Further, an mRNA-seq was performed as previously described [38,39], and expression values for each gene were normalized by the total number of reads/sample and corrected for gene length (reads/kilobasepair/million mapped reads, RPKM). Genes with RPKM > 0.1, fold-change (RAS-STCs/Normal-STCs) ≥ 1.4, and *p* ≤ 0.05 were classified as upregulated in RAS-STCs, and those with RPKM > 0.1, fold-change (RAS-STCs/Normal-STCs) ≤ 0.7 and *p* ≤ 0.05 as downregulated. To identify gene sets dysregulated at both epigenetic (MeDIP- and hMeDIP-seq) and expression (mRNA-seq) levels, these datasets were compared using GSEA. Screening analysis was run for all online available gene sets (>31,000). Results were ranked by the normalized enrichment score (NES) using a threshold of ≥+1.4 and ≤−1.4. Filtered gene sets run on the mRNA-seq and MeDIP- or hMeDIP-seq datasets were cross-compared and visualized in a scatter plot. For each quadrant of the scatter plots, the filtered gene sets with the top 10 most extreme mRNA-seq NES were extracted, and the number of apoptosis- (GO:0006915), proteolysis- (GO:0006508), and mitochondria-related (Human MitoCarta2.0 [40]) genes within them were presented.

### 2.5. Validation of MeDIP- and hMeDIP-seq/mRNA-seq Analysis

Using the ΔΔCt method on RNA isolation, cDNA synthesis, and qPCR, we compared expression levels of the candidate genes *EBF4*: *ss06930836*, *GEN1*: ss06923974, *MIS18BP1*: *ss06917788*, *PEMT*: *ss03384368*, *SGO1*: *ss06876455*, *SHARPIN*: *ss06920790*, and *SMPDL3A*: *ss06892702* between Normal- and RAS-STCs. Gene expression was normalized to GAPDH.

### 2.6. Functional Studies

To explore the impact of RAS-induced epigenetic and gene expression changes on corresponding STC integrity and function, we assessed apoptosis, proteolysis, and mitochondrial structure and function in vitro. Apoptosis was evaluated by terminal deoxynucleotidyl transferase dUTP nick-end-labeling (TUNEL) and gene expression of B-cell Lymphoma (BCL)-2 by qPCR [41,42].

Proteolysis was assessed by protein expression of ubiquitin (Cell Signaling, cat#: 58395s, 1:1000), and liquid chromatography-tandem mass spectrometry (LC-MS/MS) metabolomic analysis for amino acids. Briefly, STCs were grown in 10 cm dishes in Medium 199 with 3% FBS (Gibco BRL, Waltham, MA, USA) until optimal confluency. Cells were then treated with 1.5–2 mL cold methanol to the dish (methanol was stored in a clean bottle at −20 °C), scrapped to dislodge them from the dish’s surface, and transferred to 2 mL tubes placed on dry ice. Samples were dried and reconstituted with aTRAQ Reagent 113-labeled Standard Mix, and amino acids were separated and detected by LC-MSMS. The concentrations of amino acids were established by comparing their ion intensity (121-labeled amino acids) to their respective internal standards (113-labeled amino acids) [43].

Mitochondrial morphology was assessed by transmission electron microscopy, as previously described [6,44]. STCs were suspended overnight in Trump’s fixative solution and then examined using digital electron microscopy (Philips CM10 Transmission Electron Microscope). The number of mitochondria/cells was counted and averaged in 10 randomly selected STCs, and the mitochondrial area and matrix density were measured using ImageJ (version 1.44 for Windows) [45].

Mitochondrial superoxide production was measured in cells stained with 2 μM Mito-SOX red reagent (ThermoFisher, CA, cat#: M36008) for 30 min at 37 °C [46], whereas the membrane potential was assessed in STCs stained with tetramethylrhodamine ethyl ester (TMRE, 50 nM, ThermoFisher, CA, cat#: T669) for 20 min at 37 °C [47]. STC ATP generation (ATP/ADP ratio) was assessed in isolated mitochondria (MITO-ISO kit, Catalog#8268, ScienCell, Carlsbad, CA, USA) by colorimetric and fluorometric methods (catalog nos. ab83355 and ab83359, Abcam) [6].

### 2.7. Statistical Analysis

Statistical analysis was performed using JMP Pro14.0 (SAS) software. Results are expressed as mean ± standard deviation and considered significant for *p* ≤ 0.05. The Shapiro–Wilk test was used to test for deviation from normality. Normally and non-normally distributed data were compared using Student’s *t*-test and nonparametric methods (Wilcoxon or Kruskal–Wallis), respectively.

## 3. Results

Table 1 describes the systemic characteristics and renal function of Normal and RAS groups at the end of the study. The body weight was similar between the groups, and RAS pigs achieved a significant degree of stenosis. Systolic, diastolic, and mean arterial pressure were higher in RAS compared to Normal pigs, as were serum creatinine levels. Stenotic-kidney cortical volume, perfusion, RBF, and GFR were lower in RAS versus Normal pigs, whereas the medullary volume and perfusion were comparable between the groups.

### 3.1. STC Characterization

**Figure 1:**
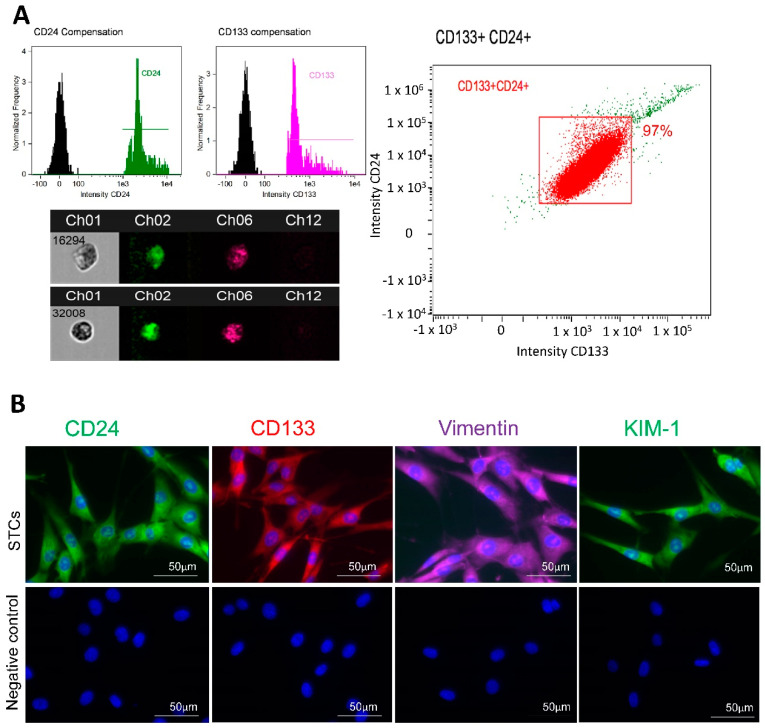
Characterization of scattered tubular-like cells (STCs) isolated from pig kidneys. (**A**): Flow cytometry analysis of isolated STCs co-expressing CD133 and CD24 (97%) and representative images of CD133+ (pink)/CD24+ (green) cells. (**B**): Immunofluorescence staining (original magnification: ×40) of swine STCs with antibodies against the surface markers CD24 (green), CD133 (red), vimentin (pink), and kidney injury molecule (KIM)-1 (green). Cells processed without primary antibodies served as negative controls.

Flow cytometry analysis showed that cultured pig STCs co-expressed CD24 and CD133 markers at a purity of 97% (Figure 1A). Immunofluorescence staining also confirmed their positivity for vimentin and KIM-1 (Figure 1B).

### 3.2. RAS Alters 5mC and 5hmC Levels in Swine STCs

**Figure 2:**
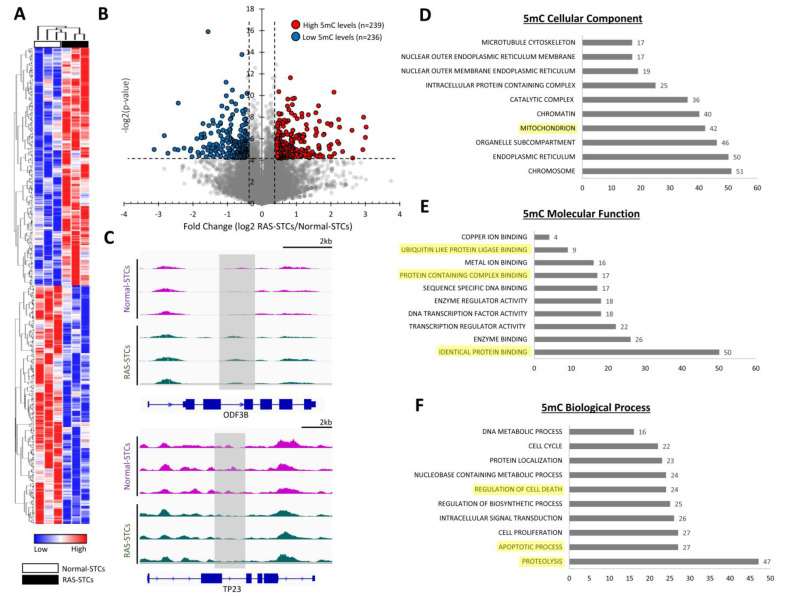
Renal artery stenosis (RAS) induces significant changes in 5-methylcytosine (5mC) levels in swine STCs. (**A**): Heat map of genes with significant changes in 5mC levels between Normal- and RAS-STCs. (**B**): Volcano plot of dysregulated 5mC genes. The vertical axis (y-axis) corresponds to the -log 2 (*p*-value) and the horizontal axis (x-axis) displays the log 2-fold change (RAS-STCs/Normal-STCs) value. Genes with higher (*n* = 239) and lower (*n* = 236) 5mC levels in RAS- versus Normal-STCs are shown with red and blue dots, respectively, while non-significant genes are shown as grey dots. A *p*-value ≤ 0.05 and fold changes ≥1.4 or ≤0.7 are indicated by black dashed lines. (**C**): Representative reads for ODF3B and TP23 genes in Normal- and RAS-STCs were visualized using Integrative Genomics Viewer. Gray rectangles indicate regions (peaks) with higher and lower 5mC levels in RAS- versus Normal-STCs. (**D**–**F**): Gene ontology (GO) analysis of cellular component (**D**), molecular function (**E**), and biological process (**F**) of genes with significant changes in 5mC levels between Normal- and RAS-STCs.

MeDIP-seq analysis identified 475 genes with significant changes in 5mC levels (239 genes with higher and 236 genes with lower 5mC levels) between RAS-STCs and Normal-STCs (fold change ≥1.4 or <0.7, *p* ≤ 0.05) (Figure 2A,B), including *ODF3B* and *TP23* (Figure 2C). GO analysis showed that genes with higher or lower 5mC levels in RAS-STCs encoded for proteins primarily located in chromosomes and organelles, such as the endoplasmic reticulum and mitochondria (Figure 2D). Proteins encoded by these genes have protein binding, enzymatic, and transcription regulator activity (Figure 2E). Analysis of their biological processes revealed that these proteins are mostly implicated in proteolysis, apoptosis, and regulation of cell death (Figure 2F).

**Figure 3:**
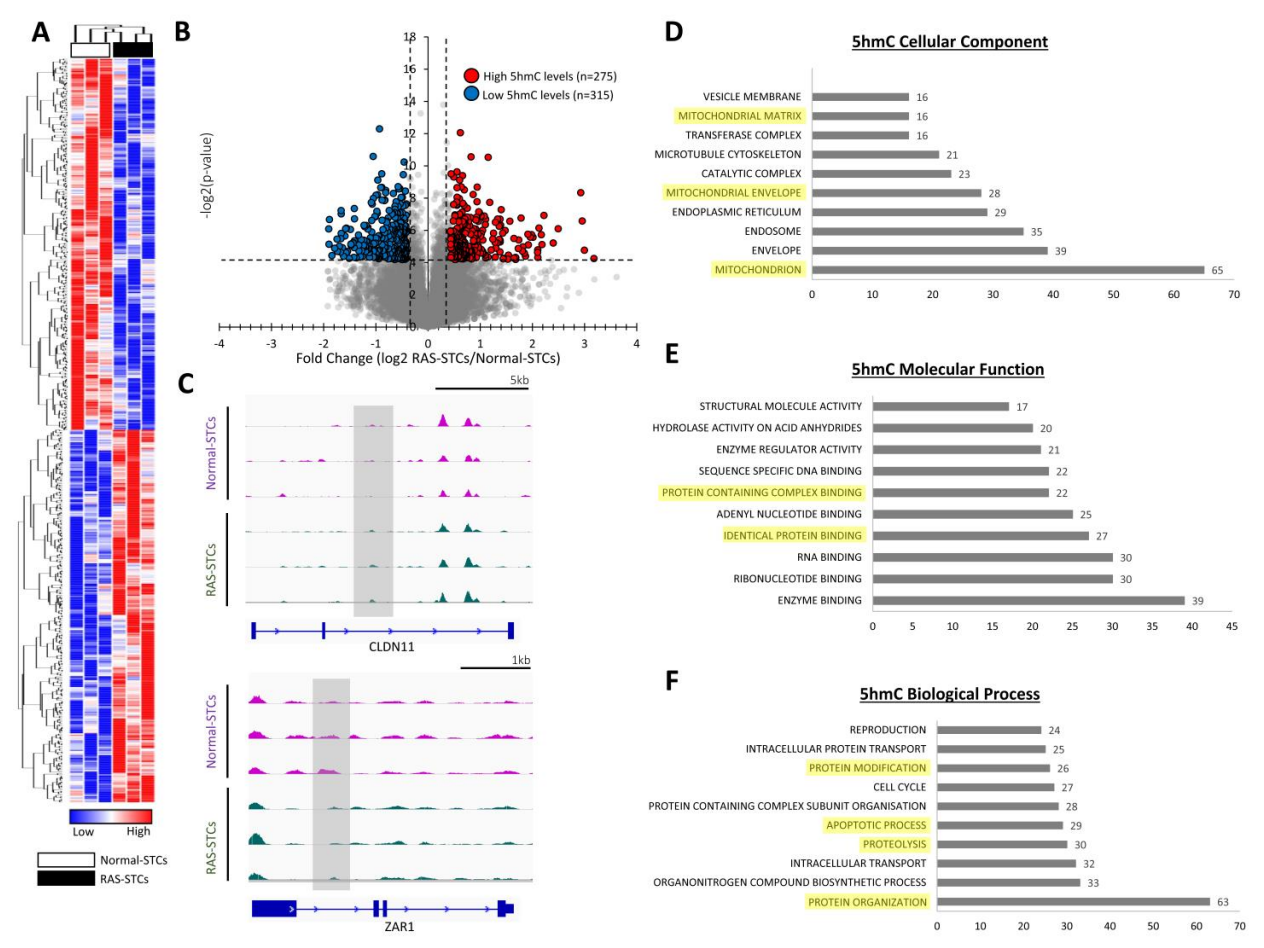
RAS induces significant changes in 5-hydroxymethylcytosine (5hmC) levels in swine STCs. (**A**): Heat map of genes with significant changes in 5hmC levels between Normal- and RAS-STCs. (**B**): Volcano plot of dysregulated 5hmC genes. Genes with higher (*n* = 275) and lower (*n* = 315) 5hmC levels in RAS- versus Normal-STCs are shown with red and blue dots, respectively. (**C**): Representative reads for CLDN11 and ZAR1 genes in Normal- and RAS-STCs. D–F: Gene ontology (GO) analysis of cellular component (**D**), molecular function (**E**), and biological process (**F**) of genes with significant changes in 5hmC levels between Normal- and RAS-STCs.

MeDIP-seq analysis revealed 590 genes with significant changes in 5hmC levels (275 genes with higher and 315 genes with lower 5hmC levels) between RAS-STCs and Normal-STCs (fold change ≥1.4 or ≤0.7, *p* < 0.05) (Figure 3A,B), including *CLDN11* and *ZAR1* (Figure 3C). Gene products were primarily located in the mitochondrion (Figure 3D), possessed enzymatic and protein binding activity (Figure 3E), and were mainly implicated in protein organization, proteolysis, and apoptosis (Figure 3F).

### 3.3. RAS Induces Changes in Gene Expression in Swine STCs

**Figure 4:**
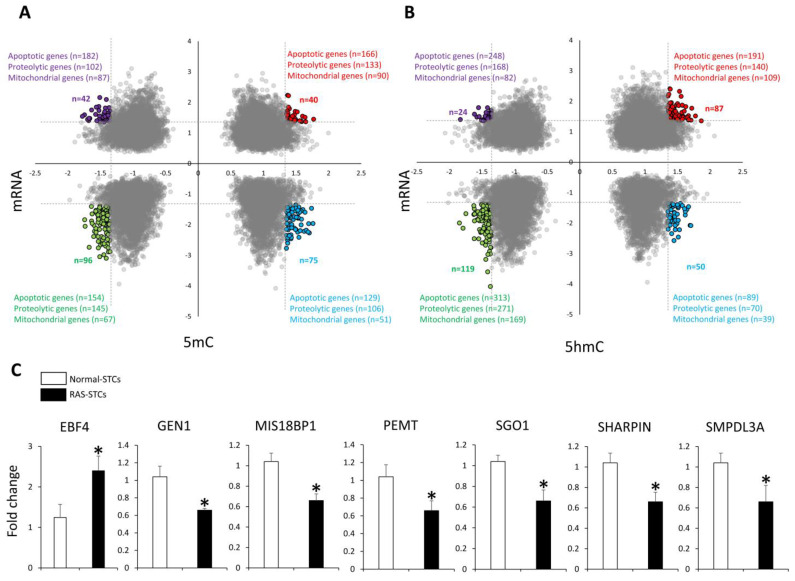
RAS-induced 5mC and 5hmC marks correlate with changes in gene expression in swine STCs. (**A**) Scatter plots of gene sets with significant overlap (5mC levels and mRNA expression) between Normal- and RAS-STCs, containing apoptotic, proteolytic, and mitochondrial genes. (**B**) Scatter plot of gene sets with significant overlap (5hmC levels and mRNA expression) between Normal- and RAS-STCs, containing apoptotic, proteolytic, and mitochondrial genes. (**C**) Expression (qPCR) of randomly selected genes dysregulated in RAS- versus Normal-STCs followed the same patterns as the mRNA-seq findings. * *p* < 0.05 vs. Normal-STCs.

Integrated 5mC-MeDIP-seq/mRNA-seq analysis identified 75 gene sets with higher 5mC and lower mRNA levels, and 42 gene sets with lower 5mC and higher mRNA levels in RAS-STCs versus Normal-STCs (Figure 4A). In contrast, there were 40 gene sets with higher and 96 with lower both 5mC and mRNA levels in RAS-STCs versus Normal-STCs.

Integrated 5hmC-MeDIP-seq/mRNA-seq analysis identified 87 gene sets with higher and 119 with lower 5hmC and mRNA levels, 50 gene sets with higher 5hmC and lower mRNA levels, and 24 gene sets with lower 5hmC and higher mRNA levels in RAS-STCs versus Normal-STCs (Figure 4B). Gene sets for which mRNA expression followed the same and opposite direction as MeDIP-seq (5mC) and hMeDIP-seq (5hmC) contained a significant number of apoptosis-, proteolysis-, and mitochondria-related genes.

The expression of randomly selected genes (*EBF4*, *GEN1*, *MIS18BP1*, *PEMT*, *SGO1*, *SHARPIN*, and *SMPDL3A*) followed the same direction as the mRNA-seq analysis (Figure 4C).

### 3.4. RAS Modulates Apoptosis, Proteolysis, and Mitochondrial Function in Swine STCs

**Figure 5:**
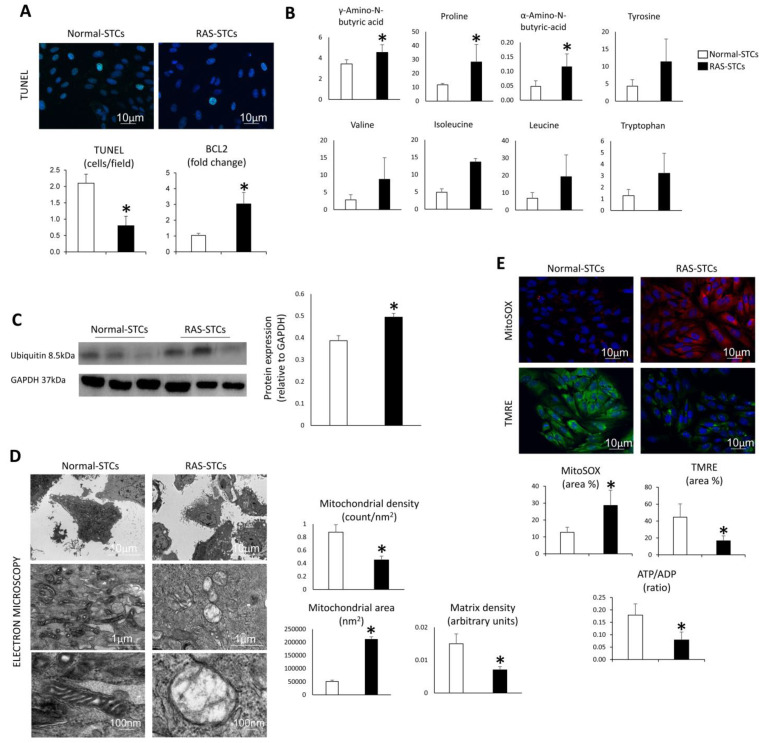
RAS alters apoptosis, proteolysis, and mitochondrial function in swine STCs. (**A**) Representative images of TUNEL staining and its quantification, as well as quantification of BCL-2 expression (qPCR) in Normal- and RAS-STCs. (**B**) Quantification of amino acid metabolites (LC-MS) in Normal- and RAS-STCs. (**C**) Protein expression of ubiquitin (Western blotting) in Normal- and RAS-STCs (Figure S1). (**D**) Representative transmission electron microscopy images and quantification of mitochondrial density, area, and matrix density in Normal- and RAS-STCs. (**E**) Mito-SOX (red) and tetramethylrhodamine ethyl ester (TMRE, green), and mitochondrial ATP generation (ATP/ADP ratio) in Normal- and RAS-STCs. * *p* < 0.05 vs. Normal-STCs.

The number of TUNEL positive STCs was lower and gene expression of BCL-2 higher in RAS-STCs compared to Normal-STCs (Figure 5A). LC-MS/MS showed higher levels of the amino acids gamma-amino-N butyric-acid, proline, alpha-amino-N butyric-acid, tyrosine, valine, leucine, isoleucine, and tryptophan in RAS- STCs versus Normal-STCs (Figure 5B), associated with higher protein expression of the proteolytic ubiquitin (Figure 5C and Figure S1). Mitochondrial and matrix density were both lower, but their area was higher in RAS-STCs compared to normal-STCs (Figure 5D). RAS-STCs exhibited a markedly higher production of mitochondrial ROS, yet mitochondrial membrane potential and ATP production were lower in RAS-STCs compared to Normal-STCs (Figure 5E).

## 4. Discussion

The current study shows that experimental RAS alters the epigenomic landscape of swine STCs, primarily involved in cellular processes associated with apoptosis, proteolysis, and mitochondrial functions. Importantly, changes in 5mC and 5hmC levels of these genes were associated with altered gene expression as well as cellular function. RAS-STCs exhibited mitochondrial structural abnormalities (swelling, loss of cristae membranes) and dysfunction (decreased membrane potential and ATP generation), decreased apoptosis, and increased proteolysis. Therefore, our observations suggest that epigenetic changes may modify the phenotype of STCs, which might account for their impaired tissue repair potency in RAS.

Repair processes in adult kidneys involve resident renal cells, such as STCs, that survive injury and dedifferentiate to acquire clonal proliferative potential [48,49]. We have previously shown that injection of Normal-STCs or their daughter extracellular vesicles into the aorta of two kidneys—1-clip mice 2 weeks after surgery improved renal function and attenuated tubular injury and fibrosis [12,50]. These renoprotective effects were blunted when we injected RAS-STCs, which have impaired proliferative potential and the ability to repair tubular epithelial cell viability in vitro [6,11]. To gain insight into the mechanisms underpinning RAS-induced STC dysfunction, we took advantage of a well-established preclinical swine model of RAS [20] and high-throughput techniques for epigenetic analysis on a genome-wide scale of STCs. We investigated two distinct epigenetic marks that play direct roles in gene transcription; 5mC, an important repressor of transcription in the genome [18] and 5hmC, which is commonly associated with transcriptional activation [19].

Our MeDIP-seq analysis revealed that RAS-STCs exhibited differential methylation in apoptotic genes, such as the B-Cell Lymphoma/Leukemia 10 (*BCL10*), which encodes a protein that contains a caspase recruitment domain and induces apoptosis, as well as Annexin A8 (*ANXA8*), which encodes a calcium-regulated phospholipid-binding protein that modulates caspase-3 and -7 activities [51]. Furthermore, we found that 5mC levels of Caspase 8 Associated Protein 2 (*CASP8AP2*), which encodes a component of the death-inducing signaling complex, and Cellular Retinoic Acid Binding Protein 1 (*CRABP1*), which regulates protein phosphatase 2A activity and facilitates apoptosis [52], were higher in RAS-STCs compared to Normal-STCs. In line with this, our hMeDIP-seq analysis found that 5hmC levels of the Transmembrane BAX Inhibitor Motif Containing 6 (*TMBIM6*), which attenuates endoplasmic reticulum stress response and apoptosis [53], were higher in RAS-STCs compared to Normal-STCs. Therefore, these observations suggest that RAS increases the methylation of pro-apoptotic and hydroxymethylation of anti-apoptotic genes in swine STCs.

RAS also induced epigenetic alterations in proteolysis-related genes in swine STCs. For example, 5mC levels of the Heat Shock Protein 90 Alpha Family Class B Member 1 (*HSP90AB1*), which encodes a chaperone protein involved in protein folding and degradation [54], were lower in RAS- versus Normal-STCs, as were levels of the Ubiquitin Conjugating Enzyme E2 V2 (*UBE2V2*), which targets abnormal or short-lived proteins for degradation [55]. Congruently, we found that 5hmC levels of the Ubiquitin Conjugating Enzyme E2 L6 (*UBE2L6*) and Ubiquitin Conjugating Enzyme E2 E2 (*UBE2E2*), were higher in RAS- versus Normal-STCs, suggesting that STCs exposed to RAS shift transcriptional repression and activation towards the cleavage of proteins into small peptides or amino acids.

Furthermore, we found that 5mC levels of mitochondrial structural and functional genes were higher in RAS-STCs compared to Normal-STCs. Among them are the Reactive Oxygen Species Modulator 1 (*ROMO1*), a mitochondrial membrane protein that modulates cellular reactive oxygen species [56], and the DNA Polymerase Gamma 2, Accessory Subunit (POLG2), which encodes the processivity subunit of the mitochondrial DNA polymerase gamma, responsible for both replication and repair of mitochondrial DNA [57]. Similarly, 5mC levels of the Acyl-CoA Synthetase Long Chain Family Member 4 (ACSL4), which plays a key role in lipid biosynthesis and fatty acid degradation [58], were higher in RAS-STCs versus Normal-STCs. In agreement, we found that 5hmC levels of several mitochondrial genes were lower in RAS-STCs compared to Normal-STCs, including the subunit of the mitochondrial oxidative phosphorylation complex I NADH:Ubiquinone Oxidoreductase Subunit B9 (*NDUFB9*), the Translocase Of Outer Mitochondrial Membrane 20 (*TOMM20*), and Pyruvate Dehydrogenase Kinase 1 (*PDK1*), which plays an important role in regulating glycolysis and mitochondrial oxidative phosphorylation by catalyzing the oxidative decarboxylation of pyruvate [59]. Thus, RAS-triggered changes in 5mC and 5hmC levels have the potential to compromise STC mitochondrial integrity and curtail their function.

To ascertain if RAS-induced epigenetic changes were associated with altered gene expression, we performed an integrated MeDIP-hMeDIP-seq/mRNA-seq analysis and identified a considerable number of gene sets for which the mRNA expression inversely correlated with the MeDIP-seq levels but directly with the hMeDIP-seq levels, including proteolytic, apoptotic, and mitochondrial genes. These observations are consistent with 5mC-induced gene repression and 5hmC-induced transcriptional activation of these genes. However, there were gene sets for which mRNA levels did not follow the expected direction of the 5mC and 5hmC findings. Therefore, other regulatory mechanisms, such as microRNAs [11] or long non-coding RNAs [60], could have played important roles in gene regulation in RAS-STCs, warranting further exploration in future studies.

Importantly, epigenetic alterations in apoptotic, proteolytic, and mitochondrial genes were associated with changes in the phenotype and function of RAS-STCs. We found that the number of apoptotic cells was lower in RAS-STCs compared to Normal-STCs, associated with increased gene expression of the pro-survival BCL-2, an integral outer mitochondrial membrane protein that blocks apoptotic cell death [61]. Protein expression of ubiquitin, which binds covalently to target proteins and marks them for proteolytic degradation [62], was higher in RAS-STCs versus Normal-STCs, associated with the accumulation of several amino acids. Proteolysis plays an important role in regulating apoptosis by modulating the cell cycle and gene expression. Indeed, proteasome inhibitors, such as lactacystin or epoximycin, are potent inducers of apoptosis in numerous cell types by preventing the degradation of specific regulatory proteins [63]. Therefore, RAS-induced alterations in proteolysis could have partly accounted for decreased apoptosis in swine STCs.

Mitochondrial density was lower, however, their area was higher in RAS-STCs compared to Normal-STCs, possibly due to the influx of water that alters the osmotic balance between cytosol and mitochondria [64,65]. Mitochondrial swelling compresses cristae membranes [66], in line with our observation of decreased matrix density in RAS-STCs. Furthermore, mitochondrial superoxide production was higher in RAS-STCs compared to Normal-STCs. Mitochondria ROS can oxidatively damage mitochondrial lipids, DNA, and respiratory protein complexes, impairing energy production [67], which is reflected in decreased mitochondrial membrane potential and ATP production. Taken together, these observations suggest that RAS-induced epigenetic and transcriptomic alterations modulate apoptosis, proteolysis, and mitochondrial function in swine STCs.

Our study has some limitations, including the early stage of RAS, lack of additional comorbid conditions, and use of relatively young animals. Nevertheless, our RAS pigs mimic the main features of human RAS (hypertension and renal dysfunction) and stenotic kidneys develop robust renal dysfunction and parenchymal injury [22,68]. The number of samples was modest for MeDIP-, hMeDIP-, and mRNA-seq studies, as often used in seq studies [69,70,71] due to the costs associated with these techniques. Evidently, this sample size sufficed to detect clear differences in 5mC, 5hmC, and gene expression levels between Normal- and RAS-STCs. It is possible that culture conditions may affect STC epigenetics and gene expression, however, given that Normal- and RAS-STCs were cultured in a similar way, RAS-induced renal ischemia and hypertension could have significantly contributed to the changes observed in RAS-STCs. We cannot be sure that the impaired repair capacity of STCs would result in the same epigenetic and mRNA changes in other kidney cells (e.g., tubular) as those observed in STCs, which might need to be addressed in future studies. Future studies are also needed to confirm these findings, with continued studying of the effect of STCs on the RAS pig kidney, and characterizing the epigenomic and gene expression profiles of human STCs in subjects with RAS.

## 5. Conclusions

In summary, our study shows that RAS induces epigenetic changes and alters the mRNA expression profile of swine STCs, dysregulating genes primarily involved in apoptosis, proteolysis, and mitochondrial function. Importantly, RAS indeed compromised the phenotype and function of swine STCs, which exhibited mitochondrial structural abnormalities and dysfunction, increased proteolysis, and decreased apoptosis. Therefore, our observations have important functional implications and support the notion that RAS-induced epigenetic changes may limit the regenerative potential of STCs, ultimately compromising this endogenous renal repair system. Our findings may also assist in developing novel approaches, such as epigenetic modifiers, to preserve the integrity and function of STCs in patients with RAS.

## Figures and Tables

**Table 1:** Systemic characteristic of normal and renal artery stenosis (RAS) pigs (*n* = 3 each) at 10 weeks.

Parameter	Normal	RAS
Body Weight (Kg)	51.5 ± 0.5	54.7 ± 5.5
Degree of stenosis (%)	0	86.7 ± 11.6 *
Systolic blood pressure (mmHg)	96.7 ± 1.5	158.3 ± 21.2 *
Diastolic blood pressure (mmHg)	71.7 ± 3.8	116 ± 18.5 *
Mean arterial pressure (mmHg)	80.5 ± 2.8	131.5 ± 19.2 *
Serum creatinine (mg/dL)	1.1 ± 0.0	1.9 ± 0.1 *
Cortical volume (mL)	104.3 ± 4.6	59.2 ± 25.8 *
Medullary volume (mL)	20.1 ± 1.0	20.6 ± 1.6
Cortical perfusion (mL/min/mL tissue)	5.6 ± 0.2	2.4 ± 0.5 *
Medullary perfusion (mL/min/mL tissue)	2.7 ± 0.3	2.5 ± 0.3
RBF (mL/min)	585.2 ± 65.8	343.9 ± 69.1 *
GFR (mL/min)	93.6 ± 6.4	56.1 ± 9.1 *

RBF: Renal blood flow, GFR: Glomerular filtration rate. * *p* ≤ 0.05 vs. Normal.

## Data Availability

The MeDIP-seq, hMeDIP-seq, and mRNA-seq data of each individual sample generated in this study are available online at https://figshare.com (accessed on 1 March 2022): Eirin, Alfonso (2022): STC MeDIPseq, hMeDIPseq, and mRNAseq. figshare. Dataset. https://doi.org/10.6084/m9.figshare.19576213.v1 (accessed on 1 March 2022).

## Supplementary Materials

The following supporting information can be downloaded at: https://www.mdpi.com/article/10.3390/cells11111803/s1, Figure S1: Protein expression and densitometric quantification of ubiquitin in Normal- and RAS-STCs.
