# Supplementary material for: Renal Ischemia Induces Epigenetic Changes in Apoptotic, Proteolytic, and Mitochondrial Genes in Swine Scattered Tubular-like Cells
Source: Cells. 2022 May 31;11(11):1803. doi: 10.3390/cells11111803 (PMC9180447; doi:10.3390/cells11111803)
Supplement: Supplementary file 1 [file cells-11-01803-s001.zip › cells-1700776-supplementary.pdf]

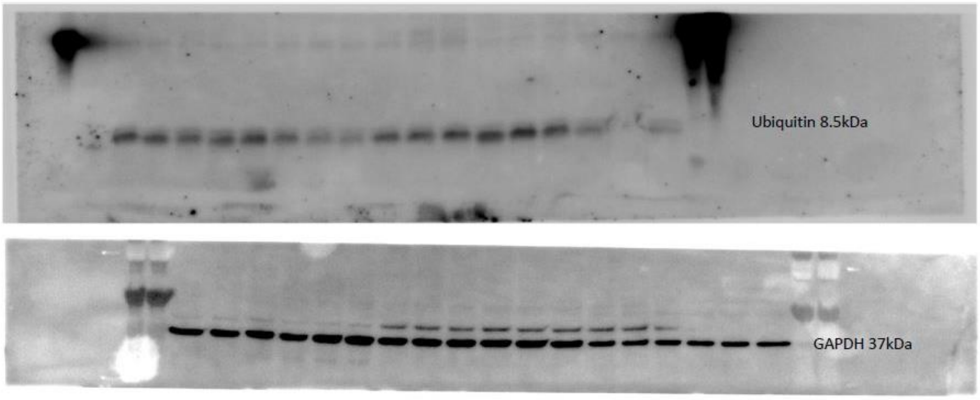

Sample	Group	Ubiquitin	GAPDH	Ubiquitin/GAPDH
1	Normal-STC	175,658	668,969	0.262580179
2	Normal-STC	305,348	662,406	0.460968047
3	Normal-STC	294,412	686,561	0.428821328
4	Normal-STC	278,729	665,551	0.418794352
5	Normal-STC	289,116	693,122	0.417121373
6	Normal-STC	314,028	696,764	0.450694927
7	Normal-STC	265,488	678,635	0.391208824
8	Normal-STC	227,463	684,242	0.332430631
9	Normal-STC	221,305	683,448	0.323806639
10	RAS-STC	277,616	680,206	0.408135183
11	RAS-STC	293,221	677,242	0.432963402
12	RAS-STC	301,930	670,152	0.450539579
13	RAS-STC	326,119	622,685	0.523730297
14	RAS-STC	332,005	610,588	0.543746356
15	RAS-STC	304,468	611,635	0.497793619
16	RAS-STC	326,119	603,629	0.54026397
17	RAS-STC	332,005	604,442	0.549275199
18	RAS-STC	304,468	608,945	0.49999261

Figure S1. Protein expression and densitometric quantification of ubiquitin in Normal- and RAS-STCs.
